# Supplementary material for: Potential Ototoxicity of Insulin-like Growth Factor 1 Receptor Signaling Inhibitors: An In Silico Drug Repurposing Study of the Regenerating Cochlear Neuron Transcriptome
Source: J Clin Med. 2023 May 16;12(10):3485. doi: 10.3390/jcm12103485 (PMC10218904; doi:10.3390/jcm12103485)
Supplement: Supplementary file 1 [file jcm-12-03485-s001.zip › jcm-2283845-supplementary.pdf]

SUPPLEMENTARY MATERIAL *for* "Potential ototoxicity of insulin-like growth factor 1 receptor-targeted therapy: An *in silico* drug repurposing study of the regenerating cochlear neuron transcriptome"

Table S1. List of genes reported to be significantly up- or down-regulated during SGN regeneration in Wu et al. [1]

	**Reason for exclusion from CLUE query**		
**Gene symbol**	**Invalid HUGO symbol or Entrez ID**	**Valid HUGO symbol or Entrez ID, but not part of BING space**	**logFC**
**Up-regulated**			
Isyna1			0.457
Mrto4			0.469
Uck2			0.469
Armc8			0.490
Abhd2			0.496
Nup93			0.505
Lmnb1			0.519
Mrps18b			0.548
Ick			0.551
Mpp6			0.554
Prkg2			0.576
Syn2		X	0.659
Asns			0.660
Abhd2			0.667
Fam171b		X	0.670
Shox2			0.708
Abhd2			0.730
Syt4		X	0.736
Gng3			0.753
Srm			0.851
Nefm			0.866
Chac1			0.984
B3gnt5		X	0.992

Spock3			1.012
Abcg5			1.171
Hba-a1	X		1.320
**Down-regulated**			
Saa3	X		-2.150
Ifi27l2a	X		-1.814
Dynlrb2		X	-1.560
Ccl5			-1.412
Chrna10			-1.325
Cep41			-1.306
Plac8			-1.256
AA467197	X		-1.231
Aoc3			-1.224
Tmem204			-1.159
Cox7a1			-1.102
Gng8		X	-1.103
Apod			-1.021
Bcl6			-0.944
Acpp			-0.938
Cd40			-0.893
Scrg1			-0.862
Scin			-0.819
Gstm2			-0.794
Chrna1			-0.776
Nme5			-0.752
Chst5			-0.734
Slc26a4			-0.718
Gstt3	X		-0.702
Flt1			-0.676
Wdr45			-0.666
Gstm2			-0.662
Pon2			-0.644
Lrrc8a		X	-0.628
Lrrc48	X		-0.616
Gstm2			-0.564
Manba			-0.563
Naprt		X	-0.545
Mb			-0.487
Rnf182		X	-0.454
Csad			-0.453

Ccdc190		X	-0.441

Abbreviations: BING, Best INFerred Genes; HUGO, Human Genome Organization; ID, identification; logFC, log fold change (in expression); SGN, spiral ganglion neuron.

Table S2. Search terms for the literature review in PubMed and ClinicalTrials.gov

Drug Name	Pharmacological Action/ Targets	Drug search terms	Otological search terms
Teprotumumab	Fully human monoclonal antibody that inhibits signaling at the insulin-like growth factor I (IGF-1) receptor and thyrotropin receptor [2]	"teprotumumab" OR "Tepezza"	"hearing" OR "hearing loss" OR "deaf" OR "deafness" OR "tinnitus" OR "vertigo" OR "ear pain" OR "ear discomfort" OR "hypoacusis" OR "ototoxic" OR "ototoxicity" OR "dizziness" OR "labyrinth" OR "audiometry" OR "audiogram"
R1507[a]	Fully human monoclonal antibody that inhibits signaling at the IGF-1 receptor and thyrotropin receptor [3]	"R1507"	
Cixutumumab	Fully human monoclonal antibody that inhibits signaling at the IGF-1 receptor [4]	"cixutumumab" OR "IMC-A12"	
Ganitumab	Fully human monoclonal antibody that inhibits signaling at the IGF-1 receptor [5]	"ganitumab" OR "AMG-479"	
Figitumumab	Fully human monoclonal antibody that inhibits signaling at the IGF-1 receptor [6]	"figitumumab" OR "CP-751871"	
Dalotuzumab	Recombinant humanized monoclonal antibody that inhibits signaling at the IGF-1 receptor [7]	"dalotuzumab" OR "MK-0646"	
Ceritinib	Tyrosine kinase inhibitor which blocks production of anaplastic lymphoma kinase (ALK) [8]	"ceritinib" OR "LDK378" OR "Zykadia"	
Ganetespib	Resorcinolic triazolone inhibitor of the molecular chaperone Hsp90, which is an upstream modifier of ALK [9]	"ganetespib" OR "STA-9090"	
Linsitinib	Small molecule inhibitor of the IGF-1 receptor and the insulin receptor [10]	"linsitinib"	

Note: [a] R1507 is also listed as the experimental name of teprotumumab but was included separately as it was not possible to determine whether alterations had occurred to the molecule prior to its market entry as teprotumumab/Tepezza.

Table S3. Characteristics of the studies and trials included in the literature review

Author/ClinicalTrial.gov ID	Study design	Treatment groups, N	Otologic AEs
**Ceritinib**			
Kim et al. [11] (NCT01283516)	Non-randomized, open-label, dose-escalation, phase I trial; 50 to 750 mg 1x daily	N=304 with tumors with genetic abnormalities in anaplastic lymphoma kinase (ALK)	500 mg: vertigo 1/10;<br>700 mg: tinnitus 1/5;<br>750 mg: vertigo 7/255, tinnitus 7/255, hearing impaired 1/255, dizziness 1/255;<br>Total: vertigo 8/304, hearing impaired 1/304, tinnitus 8/304, dizziness 42/304, serious dizziness 1/304.
NCT02040870	Non-randomized, open-label, phase I/II trial; 750 mg every 28 days	N=103 with locally advanced or metastatic non-small cell lung cancer (NSCLC) harboring a confirmed ALK rearrangement	Deafness 1/103, ear pain 1/103, dizziness 12/103
NCT02276027	Non-randomized, open-label, phase II trial; 750 mg 1x daily	N=26 with advanced NSCLC	Unilateral deafness 1/26, tinnitus 7/26, vertigo 1/26, dizziness 9/26
Soria et al. [12] (NCT01828099)	Randomized, open-label, phase III trial comparing ceritinib (750 mg/daily) vs. platinum chemotherapy	N=189 (ceritinib) and N=175 (chemotherapy) with stage IIIB/IV ALK-rearranged non-squamous NSCLC	Ceritinib: tinnitus 4/189, dizziness 22/189, serious dizziness 2/189<br>Chemotherapy: tinnitus 16/175, dizziness 17/175
Nishio et al. [13] (NCT01685138)	Open-label phase II study; 750 mg/daily	N=124 treatment-naive adults with ALK-activated NSCLC	Vertigo 1/124, dizziness 21/124, serious dizziness 3/124
Chow et al. [14] (NCT02336451)	Non-randomized, open-label phase II study, dosing not reported	N=156 with ALK-positive NSCLC metastatic to the brain and/or leptomeninges	Vertigo 7/156, dizziness 22/156, serious dizziness 1/156
Fischer et al. [15] (NCT01742286)	Open-label phase I study, various dosing	N=83 pediatric with malignancies with a ALK mutation	300 mg/fasting: dizziness 2/12, ear pain 1/12, external ear inflammation 1/12, hypoacusis 1/12, ear infection 1/12<br>510 mg/fasting: dizziness 1/13;<br>500 mg/food: dizziness 2/42, ear pain 3/42, ear infection 2/42, serious ear infection 1/42

Hida et al. [16] (NCT01685060)	Open-label phase II study, 750 mg/daily	N=140 with ALK-activated NSCLC	Dizziness 15/140
NCT02513667	Non-randomized, open-label phase II study, 10 weeks 750 mg/daily	N=13 with ALK-rearranged metastatic lung adenocarcinoma	Dizziness 2/13, vertigo 1/13
Kiura et al. [17] (NCT01828112)	Randomized, open-label phase III comparing ceritinib (750 mg/daily) vs. chemotherapy	N=115 (ceritinib) and N=113 (chemotherapy)	Ceritinib: dizziness 10/115 Chemotherapy: dizziness 6/113, severe dizziness 1/113
**Cixutumumab**			
Ferrarotto et al. [18]	Randomized open-label, phase II trial *(*10mg/kg alone or with 500mg/m^2 cetuximab every 2 weeks)	N=91 with recurrent/metastatic head and neck squamous cell carcinoma	Monotherapy: dizziness 3/47; Combination therapy: dizziness 7/44, ear pain 5/44
Chugh et al. [19] (NCT00720174)	Non-randomized, open-label, phase I trial (cixutumumab 1, 3, or 6 mg/kg with doxorubicin 75 mg/m^2)	N=29 with unresectable, locally advanced, or metastatic soft tissue sarcoma	Ear and labyrinth disorders 2/29
Dasari et al. [20] (NCT01204476)	Non-randomized, open-label, phase I trial; everolimus (10 mg daily), octreotide (20 mg every 21 days) constant, cixutumumab escalating doses of 10 mg/kg and 15 mg/kg every 21 days of a 21 day cycle	N=19 with advanced low- or intermediate-grade neuroendocrine cancer	15 mg/kg: 15 patients had baseline and end of study audiometry assessments: grade 1 change in hearing sensitivity unilaterally 4/15, bilateral changes (grade 1 and grade 2) 1/15. Changes in hearing sensitivity were not clinically significant and were identified on audiograms.
Schwartz et al. [21] (NCT01016015)	Non-randomized, open-label, phase II trial (weekly cixutumumab 6 mg/kg, IV and temsirolimus 25 mg in 6-week cycles)	N=174 with locally advanced, metastatic, or recurrent soft tissue sarcoma or bone sarcoma	Dizziness 13/174
Wilky et al. [22] (NCT01061749)	Non-randomized, open-label, dose-escalation with expansion trial cohort, phase I trial; Maximally tolerated combination dose: selumetinib 50 mg twice daily, cixutumumab 12 mg/kg every 2 weeks	N=30 with advanced solid malignancies	Dizziness 3/30, tinnitus 1/30

Lerario et al. [23] (NCT00778817)	Randomized, open-label, phase II trial; cixutumumab 10 mg/kg every 2 weeks and mitotane 2 g daily (subsequently adjusted according to serum levels/symptoms)	N=20 with inoperable recurrent, metastatic, or primary adrenocortical cancer	Tinnitus (grade I) 8/20, dizziness 5/20
NCT00520481	Non-randomized, open-label, phase II trial; 10 or 20 mg/kg every 3 weeks until disease progression or intolerable toxicity	N=41 with asymptomatic, chemotherapy-naive, metastatic, androgen-independent prostate cancer	10 mg/kg: ear disorder 2/31, vertigo 2/31, dizziness 2/31; 20 mg/kg: dizziness 1/10
NCT01413191	Non-randomized, open-label, interventional, phase II trial; 10 mg/kg on days 1 and 15 for 4 week courses	N=18 with metastatic melanoma of the eye	Ear and labyrinth disorders 4/18, tinnitus 1/18, dizziness 5/18
NCT01142388	Randomized, open-label phase II; cixutumumab 10 mg/kg on days 1 and 15 of every 28 day cycle, and paclitaxel 80 mg/m^2 on days 1, 8, and 15 of every 28 day cycle	N=44 with metastatic esophageal or gastroesophageal junction (GEJ) cancer	Control: dizziness 2/40; Treatment: dizziness 5/44
Argiris et al. [24] (NCT00955305)	Randomized, open-label phase II trial; carboplatin, paclitaxel, bevacizumab, and cixutumumab	N=82 with advanced non-squamous NSCLC	Control: dizziness 7/83; Treatment: dizziness 8/82
NCT01026623	Open-label phase I/II trial; cixutumumab and temsirolimus in varying dosages	N=16 with metastatic castration-resistant prostate cancer	Dizziness 1/16 (IMC-A12 6 mg/kg, temsirolimus 20 mg)
NCT00781911	Non-randomized, open-label phase II trial; cixutumumab 10 mg/kg every 2 weeks	N=43 with metastatic, well or moderately differentiated carcinoid or islet cell carcinoma	Control: deafness 2/31, ear discomfort 1/31, ear pain 2/31, vertigo 2/31, dizziness 12/31; ICC: Deafness 2/12, dizziness 2/12, ear discomfort 1/12, ear pain 1/12
NCT01232452	Randomized, open-label, phase II trial; Treatment: pemetrexed + cisplatin + cixutumumab for up to 4 cycles; Control: pemetrexed + cisplatin	N=85 with advanced non-squamous NSCLC	Treatment: dizziness 11/85, serious vertigo 2/85, vestibular disorder 1/85, non-serious vertigo 9/85; Control: dizziness 5/81, non-serious vertigo 4/81
NCT00778167	Open-label phase I/randomized phase II study; three dosage regimens of erlotinib hydrochloride + cixutumumab	N=18 with advanced NSCLC	Erlotinib hydrochloride 150 mg once daily with cixutumumab 15 mg/kg IV in 21-day cycles. dizziness 2/18
Yu et al. [25] (NCT01120236)	Randomized, open-label phase II study; androgen deprivation +	N=101 with hormone sensitive metastatic prostate cancer	Androgen deprivation + cixutumumab: dizziness 23/101,

	cixutumumab vs. androgen deprivation		tinnitus 6/101; Androgen deprivation: dizziness 9/104, tinnitus 1/104.
NCT00870870	Randomized, open-label Phase II trial; C1: gemcitabine + carboplatin + cetuximab (GCC); C2: gemcitabine + cisplatin + cetuximab (GCiC); TX1: GCC + cixutumumab; TX2: GCiC + cixutumumab	N=31 with advanced/metastatic NSCLC	(C1) Dizziness 1/4; (C2) ear discomfort 2/29, hypoacusis 1/29, tinnitus 5/29, dizziness 8/29; (TX2): ear discomfort 1/25, hypoacusis 2/25, tinnitus 2/25, balance disorder 3/25, dizziness 4/25.
Higano et al. [26] (NCT00785538)	Open-label phase I study	N=24 with advanced solid tumors	Dizziness 3/24
Hussain et al. [27] (NCT00683475)	Randomized, open-label phase II study; C: cixutumumab + mitoxantrone + prednisone; R: ramucirumab + mitoxantrone + prednisone	N=66 with metastatic prostate cancer	C: dizziness 9/66; R: dizziness 12/66
NCT01160458	Open-label phase II study; 20 mg/kg	N=20 with mesothelioma	Dizziness 5/20
NCT00887159	Randomized, open-label phase II study; Arm A: cisplatin + etopside; Arm B: cisplatin + etopside + vismodegib; Arm C: cisplatin + etopside + cixutumumab	N=106 with extensive small cell lung cancer	Arm C: Serious dizziness 1/52, hearing impaired 2/52, tinnitus 6/52, dizziness 13/52; Arm A: Tinnitus 4/53, dizziness 8/53; Arm B: Hearing impaired 4/53, tinnitus 5/53, serious tinnitus 1/53, dizziness 9/53
NCT00668148	Open-label phase II study; 10 mg/kg	N=111 with advanced or metastatic soft tissue and Ewing's sarcoma	Dizziness 6/111
NCT00617708	Randomized, open-label phase I and II study; TX: erlotinib + gemcitabine + cixutumumab; Control: erlotinib + gemcitabine	N=123 with metastatic pancreatic cancer	Treatment: auditory/ear-other AE 2/66, serious dizziness 1/66, dizziness 10/66; Control: dizziness 7/57
NCT00728949	Randomized, open-label phase II study; Dual Tx: cixutumumab and schedule of last antiestrogen therapy to which their disease became refractory; Mono Tx: cixutumumab	N=93 with advanced/metastatic breast cancer	Dual Tx: dizziness 8/56; Mono Tx: dizziness 3/37

NCT00503685	Randomized, open-label phase II study; TX1: 10 mg/kg IMC-A12; TX2: cetuximab 500 mg/m^2 followed by 10 mg/kg IMC-A12; TX3: cetuximab 500 mg/m^2 followed by 10 mg/kg IMC-A12	N=64 with metastatic colorectal cancer	TX1: dizziness 3/23; TX2: dizziness 2/21; TX3: dizziness 1/20
NCT00906373	Non-randomized, open-label phase II study; (1*)* 10 mg/kg cixutumumab; (2) 20 mg/kg cixutumumab	N=47 with advanced hepatocellular carcinoma	(1) Hearing impaired 1/6; (2) Ear discomfort 5/41
NCT00986674	Randomized, open-label phase II study; Arm 1: carboplatin + paclitaxel + cetuximab; Arm 2: carboplatin + paclitaxel + cixutumumab; Arm 3: carboplatin + paclitaxel + cetuximab + cixutumumab	N=90 with advanced NSCLC	Arm 1: dizziness 3/44; Arm 2: dizziness 6/42; Arm 3: dizziness 5/48
NCT00617734	Randomized, open-label phase II study; Mono: cixutumumab 10 mg/kg; Dual: cixutumumab 10 mg/kg + cetuximab 500 mg/m^2	N=97 with metastatic squamous cell carcinoma head and neck cancer	Monotherapy: dizziness 3/47; Dual therapy: dizziness 6/44, ear pain 5/44
**Dalotuzumab**			
Rugo et al. [28] (NCT01605396)	Randomized, open-label, phase II trial comparing ridoforolimus 10 mg QD 5 ×/week + dalotuzumab 10 mg/kg/week + exemestane 25 mg/day (R/D/E), vs. ridaforolimus 30 mg QD 5 ×/week + exemestane 25 mg/day (R/E)	N=40 (R/D/E) and N=40 (R/E) with postmenopausal, high-proliferation (Ki67 index staining ≥15%), ER+ breast cancer (BC) that progressed after nonsteroidal aromatase inhibitor	R/D/E: Grade 1 hearing loss 1/39, dizziness 1/39<br>R/E: Grade 1 hearing loss 1/40, dizziness 6/40
Ellis et al. [29] (NCT00869752)	Non-randomized, open-label, phase I-II trial of two doses of dalotuzumab (5 or 10 mg/kg IV weekly) + cisplatin (25 mg/m^2) and etoposide (100 mg/m^2) every 21 days	N=12 with chemotherapy-naïve advanced NSCLC	Hearing loss 2/12, one of grade 3 that was attributed to cisplatin
Huang et al. [30] (NCT00799240)	Randomized, open-label, parallel assignment, phase II trial of cisplatin + pemetrexed with or without dalotuzumab	N=12 (dalotuzumab) and N=14 (no dalotuzumab) with stage IV non-squamous lung cancer	Pemetrexed + cisplatin: grade 2 tinnitus 4/14

			Dalotuzumab with pemetrexed + cisplatin: grade 2 tinnitus 3/12, grade 3 hearing toxicity 1/12
Brana et al. [31] (NCT01243762)	Non-randomized, open label phase I study of 7 dose combinations of dalotuzumab, MK-0752, or ridaforolus	N=47 with advanced cancer	Dalotuzumab 7.5 mg/kg + MK-0752 1800 mg: dizziness 1/4;<br>Dalotuzumab 10 mg/kg + MK-0752 1800 mg: ear discomfort: 2/13, hearing impaired 1/13, tinnitus 1/13;<br>Dalotuzumab 10 mg/kg + MK-2206 135 mg: dizziness 3/8;<br>Dalotuzumab 10 mg/kg + MK-2206 150 mg: dizziness 1/10, ear discomfort 1/10, middle ear effusion 1/10, vertigo 1/10;<br>Dalotuzumab 10 mg/kg + MK-2206 200 mg: dizziness 1/3
Sclafani et al. [32] (NCT00614393)	Randomized, quadruple blinded phase II/III study of (1) dalotuzumab 10mg/kg Q1W; (2) dalotuzumab 15 mg/kg/7.5 mg/kg Q2W; (3) dalotuzumab 10 mg/kg Q1W; (4) dalotuzumab 15 mg/kg/7.5 mg/kg Q2W versus placebo + cetuximab + irinotecan	N=556 with metastatic colorectal cancer (CRC)	(1) dizziness 16/180, serious deafness bilateral 2/180;<br>(3) deafness 1/10;<br>(4) dizziness 21/180, ear infection 1/180;<br>Control: dizziness 21/178
NCT01609231	Randomized, open-label phase IIA study comparing dalotuzumab + irinotecan vs. cetuximab + irinotecan	N=11 with metastatic CRC	Dalotuzumab + irinotecan: dizziness 1/6, ear discomfort 1/6, vertigo 1/6
NCT00694356	Non-randomized, open-label phase I study of 3 doses of dalotuzumab	N=15 with relapsed or refractory locally advanced or metastatic solid tumors	5 mg/kg: dizziness 1/6;<br>15 mg/kg/7.5 mg/kg: ear congestion 1/6
NCT00635778	Non-randomized, open-label phase I study of 7 doses of dalotuzumab	N=50 with relapsed or refractory locally advanced or metastatic cancers	2.5 mg/kg: vertigo 1/3;<br>10 mg/kg: deafness 1/3, ear infection 1/3;<br>15 mg/kg: dizziness 2/21
Moran et al. [33] (NCT00654420)	Randomized, open-label phase I/IIA study of (1) dalotuzumab 5 mg/kg + erlotinib; (2) dalotuzumab 10 mg/kg + erlotinib;	N=95 with recurrent NSCLC	(2) dizziness 1/16;<br>(3) dizziness 5/37;<br>(4) vertigo 2/38, dizziness 3/38

	(3):dalotuzumab 10 mg/kg + erlotinib; or (4) erlotinib only		
**Figitumumab**			
Becerra et al. [34] (NCT00560560)	Non-randomized, open-label, phase II trial; A: 20 mg/kg in 3 week cycles; B: 30 mg/kg	N=83 with metastatic CRC that was refractory to ≥2 systemic therapies	A: cerumen impaction 1/85, deafness 2/85, hypoacusis 2/85, tinnitus 1/85, vertigo 1/85, serious dizziness 1/85, dizziness 3/85; B: serious bilateral deafness 1/83, deafness 2/83, hypoacusis 2/83, deafness bilateral 1/83, deafness unilateral 1/83, motion sickness 1/83, ear pain 1/83, dizziness 3/83
Langer et al. [35] (NCT00596830)	Randomized, open-label, phase III trial; figitumumab (F) + paclitaxel and carboplatin; Control: paclitaxel and carboplatin alone	N=671 with stage IIIB/IV or recurrent NSCLC disease with non-adenocarcinoma histology	Tx: dizziness 37/338, serious dizziness 1/338, vertigo 1/338; Control: dizziness 32/333
Yin et al. [36]	Non-randomized, open-label, phase I trial; single dose of 10 or 20 mg/kg	N=28 healthy males	3 cases of tinnitus were reported at 10 mg/kg but none at 20 mg/kg. Tinnitus resolved in 2 participants within 21 and 171 days, respectively, from the start of symptoms; in the third participant, Tinnitus resolved within 25 days in one ear but was ongoing in the other ear 227 days after dosing. Two of the 3 participants with tinnitus also had an abnormal audiogram of mild severity.
NCT00729833	Non-randomized, open-label, sequential cohort, dose escalation, phase I trial; A: figitumumab (F) 10 mg/kg on day 1 of each cycle 3-week + sunitinib (S) 25 mg; B: F 10 mg/kg + S 37.5 mg; C: F 20 mg/kg + S 25 mg; D: F 20mg/kg + S 25 mg schedule 2/1	N=45 with advanced solid tumors	A: deafness bilateral 1/20, tinnitus 2/20, dizziness 3/20; B: dizziness 1/7; C: ear infection 1/12; D: tinnitus 1/6.
NCT00560573	Non-randomized, open-label, phase I trial; figitumumab 6 mg/kg (A), 10 mg/kg (B), 20 mg/kg (C,	N=45 with advanced NSCLC	A: deafness 1/6, hypoacusis 1/6, ototoxicity 1/6, tinnitus 2/6; C: ototoxicity 3/6, tinnitus 2/6; D: deafness 1/10, motion sickness

	D, E, F) + cisplatin + gemcitabine/pemetrexed		1/10, ototoxicity 3/10, tinnitus 1/10; E: hypoacusis 1/7, ototoxicity 2/7, tinnitus 1/7, dizziness 4/7; F: deafness 1/13, hypoacusis 2/13, ototoxicity 3/13, tinnitus 2/13
Scagliotti et al. [37](NCT00673049)	Randomized, open-label, phase III trial; A: IV 20 mg/kg figitumumab on days 1 and 2 in cycle 1 and on day 1 every 3 weeks, in combination with erlotinib 150 mg daily; B: erlotinib 150 mg daily; C erlotinib, then figitumumab 20 mg/kg as a single-agent therapy in 3-week cycles on study Days 1 and 2 in Cycle 1, and on Day 1 every 3 weeks.	N=662 with advanced NSCLC with non-adenocarcinoma histology	A: serious bilateral deafness 1/289, deafness 1/289, neurosensory deafness 1/289, unilateral deafness 2/289, ear disorder 1/289, ear pain 1/289, hearing impaired 1/289, tinnitus 3/289, tympanic membrane perforation 1/289, vertigo 3/289, positional vertigo 1/289, vestibular disorder 1/289, dizziness 16/289; B: ear pain 1/290, tinnitus 2/290, dizziness 4/290; C: ear pain 1/83, hearing impaired 1/83, dizziness 3/83
NCT00976508	Non-randomized, open-label, safety and tolerability, phase I trial; A: IV figitumumab 20 mg/kg every 3 weeks up to 1 year + pegvisomat 10 mg B: IV figitumumab 20 mg/kg every 3 weeks up to 1 year + pegvisomat 20 mg.	N=23 with advanced solid tumors	A: ear discomfort 1/17, sudden hearing loss 1/17, dizziness 2/17; B: ear discomfort 2/6, hearing impaired 1/6
Olmos et al. [38] (NCT00474760)	Non-randomized, open-label phase I study; T1: figitumumab (F) 3 mg/kg; T2: F 6 mg/kg; T3: F 10 mg/kg; T4: F 20 mg/kg; T5: F 20 mg/kg RP2D; T6: F 20 mg/kg recommended phase II dose (RP2D) ACC+ sarcoma; T7: F 20 mg/kg recommended phase II dose (Ewings sarcoma).	N=65 with advanced solid tumors	T2: dizziness 1/3; T5: dizziness 2/13; T6: dizziness 4/29; T7: ear congestion 1/11, ear pain 2/11, tinnitus 1/11, dizziness 1/11
NCT00147537	Randomized, open-label phase Ib/II; T1: CP-751,871 0.05 mg/kg + paclitaxel + carboplatin (P+C); T2: CP-751,871 0.1 mg/kg + P+C;	N=300 with advanced NSCLC	T1: dizziness 2/3; T5: dizziness 1/3; T6: tinnitus 1/3; T7: ear disorder 1/17, tinnitus 1/17, vertigo 1/17, dizziness 1/17; T8: tinnitus

	T3: CP-751,871 0.8 mg/kg + P+C; T4: CP-751,871 1.5 mg/kg + P+C; T5: CP-751,871 3 mg/kg + P+C; T6: CP-751,871 6 mg/kg + P+C; T6: CP-751,871 10 mg/kg + P+C; T7: CP-751,871 20 mg/kg + P+C; T8: CP-751,871 + P+C + erlotinib; T9: CP-751,871 + P+C; T10: P+C; T11: P+C + additional CP-751,871		1/7, dizziness 3/7; T9: ear congestion 1/17, hearing impaired 1/17, hypoacusis 1/17, ear infection 1/17, dizziness: 4/17; T10: ear disorder 1/163, hypoacusis 7/163, tinnitus 8/163, vertigo 3/163, deafness 6/163, ear discomfort 1/163, ear pain 3/163, ear infection 1/163, dizziness 27/163, serious dizziness 1/163; T11: tinnitus 1/56, dizziness 4/56; T12: ear disorder 1/22, tinnitus 1/22, vertigo 1/22, cerumen impaction 1/22, deafness 2/22, vestibular disorder 1/22, dizziness 4/22. G: dizziness 46/300, serious dizziness 1/300, ear infection 2/300, vestibular disorder 1/300, ear pain 3/300, ear discomfort 1/300, deafness 8/300, cerumen impaction 1/300, vertigo 5/300, tinnitus 13/300, hypoacusis 8/300, hearing impaired 1/300, ear disorder 3/300, ear congestion 1/300
NCT00977561	Randomized, open-label phase II study; figitumumab + cisplatin or carboplatin + etoposide; Ctr: cisplatin or carboplatin + etoposide	N=9 with extensive stage small cell lung cancer	Tx: deafness: 1/5; Ctr: tinnitus 1/4, dizziness 1/4
**Ganetespib**			
NCT01111838	Non-randomized, open-label, single-arm, phase II trial (200 mg infusion, 1x week for 3 weeks)	N=15 with refractory metastatic CRC	Tinnitus 1/15, dizziness 1/15
NCT01039519	Non-randomized, open-label phase II study (200 mg infusion, 1x week for 3 weeks)	N=27 with metastatic/unresectable gastrointestinal stromal tumor (GIST)	Ear discomfort 1/27, dizziness 4/27, labyrinthitis 1/27
NCT01798485	Randomized, open-label phase III study comparing ganetespib (150 mg/m^2) + docetaxel (75 mg/m^2) vs. docetaxel (75 mg/m^2)	N=338 (ganetespib + docetaxel) and N=342 (docetaxel) with advanced NSCLC	ganetespib + docetaxel: vertigo 1/338, dizziness 24/338 docetaxel: dizziness 21/342

NCT01200238	Non-randomized, open-label phase II study comparing 2 dosages (150 vs. 200 mg/m^2 infusions)	N=17 with metastatic ocular melanoma	150 mg/m^2: dizziness 2/10, ear and labyrinth disorder 1/10
Meehan et al. [39] (NCT02192541)	Open-label phase I study of ganetespib 100 mg/m^2 + ziv-aflibercept 4 mg/kg or ganetespib 100 mg/m^2 + ziv-aflibercept 3 mg/kg	N=5 with advanced gastrointestinal carcinomas, NSCLC, urothelial carcinomas, or sarcomas	Ganetespib 100 mg/m^2 + ziv-aflibercept 3 mg/kg: dizziness 1/3
NCT01962948	Non-randomized, open-label phase I/II study of three combinations: (1) 100 mg/m^2 ganetespib + 80 mg/m^2 paclitaxel; (2) 125 mg/m^2 ganetespib + 80 mg/m^2 paclitaxel; (3) 150 mg/m^2 ganetespib + 80 mg/m^2 paclitaxel	N=12 with recurrent, platinum-resistant ovarian, fallopian tube, or primary peritoneal cancer	(1) hearing loss 1/3;<br>(3) hearing loss 1/6, tinnitus 1/6, vertigo 1/6
NCT01227018	Open-label phase II study (175 mg infusion, 1x week for 3 weeks)	N=14 with metastatic pancreas cancer	Dizziness 1/14
**Ganitumab**			
Strosberg et al. [40] (NCT01024387)	Non-randomized, open-label, phase II trial (18 mg/kg over 60 min every 3 weeks)	N=60 with metastatic low- and intermediate-grade carcinoid or pancreatic neuroendocrine tumors	Hearing impairment 1/60, ear or labyrinth disorders 1/60, dizziness 3/60
NCT01708161	Non-randomized, open-label, single arm, phase Ib/II trial (various doses of alpelisib + 12 mg/kg ganitumab)	N=46 with *PIK3CA* mutated or amplified solid tumors, *PIK3CA* mutated or amplified HR+ BC	Alpelisib 350 mg + ganitumab 12 mg/kg: ear congestion 1/10, hearing impairment 1/10;<br>Alpelisib 300 mg + ganitumab 12 mg/kg (in HR+ BC): ear congestion 1/10, ear pain 1/10, tinnitus 1/10;<br>All: dizziness 11/46.
NCT00719212	Non-randomized, open-label, phase II trial (18 mg/kg on day 1 of each 21-day cycle)	N=61 with recurrent platinum-sensitive ovarian epithelial (including fallopian tube and primary peritoneal) carcinoma failing frontline chemotherapy	Deafness 1/61, dizziness 6/61
NCT00718523	Randomized, double-blinded, placebo controlled, phase II trial of (1) paclitaxel/carboplatin chemotherapy vs. (2) ganitumab +	N= 77 (1) and N=88 (2) with debulked stage III and IV ovarian epithelial (including fallopian tube and primary peritoneal) carcinoma	(1) deafness neurosensory 1/77, tinnitus 4/77, vertigo 4/77, dizziness 15/77;

	paclitaxel/carboplatin chemotherapy		(2) tinnitus 3/88, vertigo 8/88, serious dizziness 1/88, dizziness 9/88
**Teprotumumab**			
Douglas et al. [2] (NCT03298867)	Randomized, double-masked, placebo-controlled, phase III trial; 8 infusions, one every 3 weeks; initial dose 10 mg/kg, followed by 20 mg/kg for the remaining seven infusions	N=83 with Graves disease, active, moderate to severe thyroid eye disease (TED) and ocular symptoms (n=41 in treatment group)	Hearing impairment 5/41 (2x hypoacusis, resolved; 1x deafness, resolved; 1x autophony, resolved; 1x mild patulous eustachian tube, resolved); 32F had hypoacusis (mild) - onset d75, resolved d76; 66M had hypoacusis (moderate) - onset d108, resolved d 283, d167 peaked tympanometry and hearing threshold between 35-55 pantonal R and 35-70 dB pantonal L, moderate hearing impairment R, mild hearing impairment L; 79F had deafness (moderate) - onset d134, resolved d337, d166 peaked tympanograms in normal pressure range and bilat symmetrical pantonal hearing loss, d253 improvement; 60F had autophony (mild) - onset d84, resolution around 4 months after 8th infusion, d29 ear discomfort + dizziness (d29-51), oropharyngeal pain d81-87; 39F had Eustachian tube patulous (mild), onset d153, resolved d304, rhinorrhea d151-152, periodontitis d154-ongoing at last follow up; dizziness 3/41
Smith et al. [41] (NCT01868997)	Randomized, double-masked, placebo-controlled, phase II trial; 8 infusions, one every 3 weeks; initial dose 10 mg/kg, followed by 20 mg/kg for the remaining 7 infusions	N=87 with active TED (n=42 in treatment group)	Hearing impairment 3/42 (1x unilateral hearing impairment onset 16 weeks after end of Tx, 1x mild bilateral hearing impairment (resolved), 1x tinnitus in a patient with history of tinnitus); 59F had hyperacusis: reported d112 that her voice echoed when she spoke and

			has occasional bilateral tinnitus with brief dizziness, audiology: d112 R ear w mild hearing loss at 6kHz, L w mild conductive to mixed hearing loss at 4-8kHz, excellent word recognition bilateral, normal tympanograms, d203 stable from prior evaluation; 43F had Eustachian tube dysfunction (moderate), deafness unilateral (moderate), medical history of chronic sinusitis, d274 moderate mixed hearing loss with possible recruitment L, R normal, d497 overall improvement; 60M had deafness (mild) - medical history of intermittent tinnitus after exposure to loud noise, d222 96% word recognition R, 92% L; patient suffering from tinnitus with handicapping moderate high frequency sensorineural bilateral hearing loss, hearing aid eval recommended
Xin et al. [42] (NCT03461211)	Non-randomized, open-label, phase III extension trial; 8 infusions, one every 3 weeks; initial dose 10 mg/kg, followed by 20 mg/kg for the remaining seven infusions	N=51 with active TED	Five patients experienced 6 events of hearing impairment; ear discomfort 4/51, autophony 1/51, hypoacusis 3/51, tinnitus 2/51, vertigo 2/51, deafness 1/40 (occurred in follow up period), dizziness 1/37
Sears et al. [43]	Prospective observational case series; 8 infusions, one every 3 weeks; initial dose 10 mg/kg, followed by 20 mg/kg for the remaining seven infusions	N=27 with TED	22/27 patients developed new or worsening otologic symptoms after an average of 3.8 teprotumumab doses; Ear plugging, fullness, and pressure 13/27 (10/11 resolved), muffled hearing, hearing loss, and diminished word recognition 11/27 (5/11 resolved), tinnitus and ear popping 10/27 (10/10 resolved),

			autophony 7/27 (5/6 resolved). Three symptomatic patients lost to follow-up. 13/19 complete resolution of otologic symptoms, 6/19 persistence of at least one otologic symptom. Patients with persistent hearing loss received audiometric testing, showing decreased hearing thresholds. 4/6 required hearing aids
NCT00773383	Randomized, open-label study; teprotumumab 9 mg/kg once/week + erlotinib 150 mg once daily	N=34 with stage IIIB/IV NSCLC	Vertigo 2/34
Ramalingam et al. [44] (NCT00760929)	Randomized, double blinded, placebo controlled study; T1: teprotumumab 9 mg/kg; T2: teprotumumab 16 mg/kg; Control 1: placebo 9 mg/kg; Control 2: placebo 16 mg/kg	N=116 with advanced NSCLC	T1: dizziness 5/59; T2: dizziness 2/57; Ctr1: dizziness 4/26; Ctr2: dizziness 3/29
Pappo et al. [3] (NCT00642941)	Non-randomized, open label phase II study; teprotumumab 9 mg/kg once weekly until disease progression, intercurrent illness, unacceptable toxicity, prolonged (2-week) time off treatment, withdrawal, loss to follow-up, investigator decision, or death	N=317 with various cancers	Ear pain 6/317, tinnitus 5/317, ear discomfort 5/317, dizziness 14/317
NCT00796107	Open-label study; 2.5 mg letrozole + 16 mg/kg teprotumumab	N=6 with advanced BC	Vertigo 1/6, dizziness 1/6.

Table S4. Clinical trial and observational real-world data on otologic AEs in patients treated with teprotumumab

Clinical Trial/ Publication	Patients treated with teprotumumab, n	Reported Otologic AEs, n (%)				Patients with otologic AEs, n (%)
		Hearing	Tinnitus	Other	Total AEs	
Clinical trial, pre-approval						
NCT01868997, Smith et al. (2017) [41]	43	3 (7%)	2 (4.7%)	0 (0%)	5 (11.6%)	3 (7%)
NCT03298867, Douglas et al. (2020) [2]	41	3 (7.3%)	0 (0%)	2 (4.9%)	5 (12.2%)	5 (12.2%)
Clinical trial, post-approval						
NCT03461211	51	4 (7.8%)	2 (3.9%)	5 (9.8%)	11 (21.6%)	5 (9.8%)
Observational real-world						
Sears et al. (2022) [43]	27	11 (40.7%)	10 (37%)	20 (74.1%)	41 (151.9%)	22 (81.5%)

Caption: NCT01868997 and NCT03298867 were used in teprotumumab's United States FDA approval application (application number 761143Orig1s000 [45]). Combined, the two trials have reported 10 events of new or worsening hearing impairment, tinnitus, or other otologic AEs (including deafness, eustachian tube dysfunction, hyperacusis, hypoacusis, tinnitus, ear discomfort, and autophony) in 84 teprotumumab-treated patients, representing an incidence of 11.9%. The combined prevalence of otologic AEs in these trials was 9.5% (8/84 patients). NCT03461211 reported 11 otologic AEs in 51 teprotumumab-treated patients, representing an incidence of 21.6%, and a prevalence of 9.8% (5 of 51 patients). In an observational study of patients receiving teprotumumab in real-world clinical practice (Sears et al.), 22 of 27 patients were reported as having a total of 41 new or worsening otologic AEs (81.5% prevalence), an incidence of 151.9%. Abbreviations: AE, adverse event; FDA, Food and Drug Administration.

Fig S1. Results of the risk of bias analysis for the clinical trials included in the meta-analysis

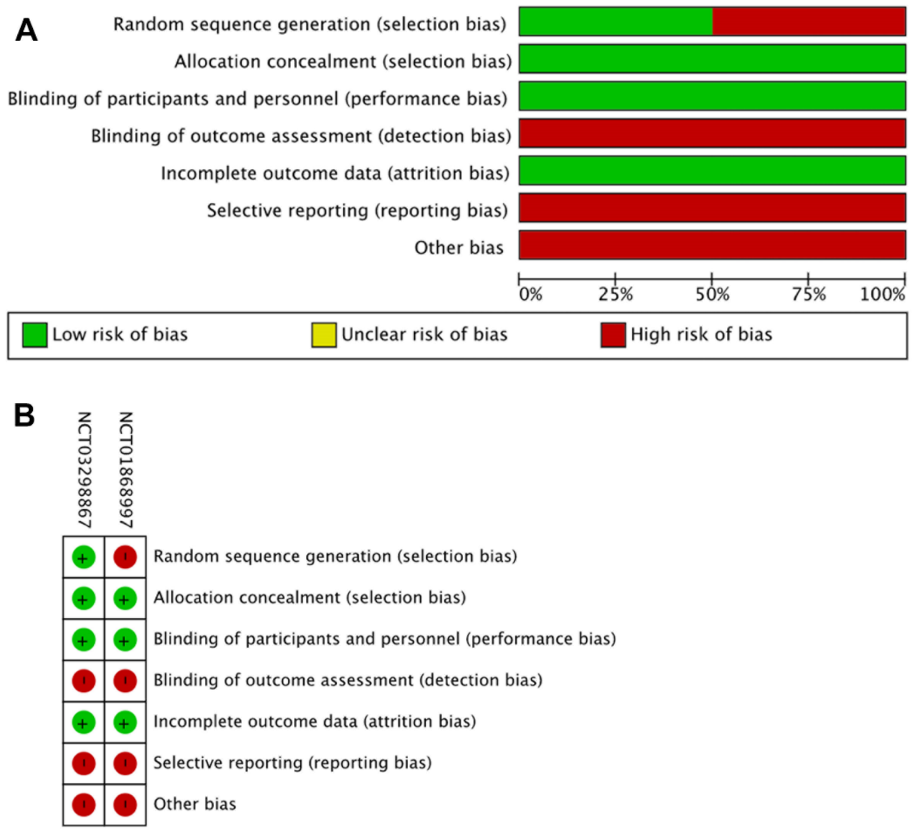

Caption: The risk of bias analysis of the two placebo-controlled clinical trials of teprotumumab for thyroid eye disease included in the meta-analysis (ClinicalTrials.gov Identifiers: NCT01868997 and NCT03298867), overall (A) and for each trial (B). There was a high risk of bias related to blinding of outcome assessment data, selective reporting bias, and other bias in both trials.

REFERENCES

1. Wu, C.C.; Brugeaud, A.; Seist, R.; Lin, H.C.; Yeh, W.H.; Petrillo, M.; Coppola, G.; Edge, A.S.B.; Stankovic, K.M. Altered expression of genes regulating inflammation and synaptogenesis during regrowth of afferent neurons to cochlear hair cells. *PLoS One* **2020**, *15*, e0238578.
2. Douglas, R.S.; Kahaly, G.J.; Patel, A.; Sile, S.; Thompson, E.H.Z.; Perdok, R.; Fleming, J.C.; Fowler, B.T.; Marcocci, C.; Marinò, M.; et al. Teprotumumab for the treatment of active thyroid eye disease. *N Engl J Med* **2020**, *382*, 341-352.
3. Pappo, A.S.; Patel, S.R.; Crowley, J.; Reinke, D.K.; Kuenkele, K.P.; Chawla, S.P.; Toner, G.C.; Maki, R.G.; Meyers, P.A.; Chugh, R.; et al. R1507, a monoclonal antibody to the insulin-like growth factor 1 receptor, in patients with recurrent or refractory Ewing sarcoma family of tumors: results of a phase II Sarcoma Alliance for Research through Collaboration study. *J Clin Oncol* **2011**, *29*, 4541-4547.
4. McKian, K.P.; Haluska, P. Cixutumumab. *Expert Opin Investig Drugs* **2009**, *18*, 1025-1033.
5. Yee, D.; Isaacs, C.; Wolf, D.M.; Yau, C.; Haluska, P.; Giridhar, K.V.; Forero-Torres, A.; Jo Chien, A.; Wallace, A.M.; Pusztai, L.; et al. Ganitumab and metformin plus standard neoadjuvant therapy in stage 2/3 breast cancer. *NPJ Breast Cancer* **2021**, *7*, 131.
6. Langer, C.J.; Novello, S.; Park, K.; Krzakowski, M.; Karp, D.D.; Mok, T.; Benner, R.J.; Scranton, J.R.; Olszanski, A.J.; Jassem, J. Randomized, Phase III trial of first-line figitumumab in combination with paclitaxel and carboplatin versus paclitaxel and carboplatin alone in patients with advanced non–small-cell lung cancer. *J Clin Oncol* **2014**, *32*, 2059-2066.
7. Scartozzi, M.; Bianconi, M.; Maccaroni, E.; Giampieri, R.; Berardi, R.; Cascinu, S. Dalotuzumab, a recombinant humanized mAb targeted against IGFR1 for the treatment of cancer. *Curr Opin Mol Ther* **2010**, *12*, 361-371.
8. Shaw, A.T.; Kim, D.W.; Mehra, R.; Tan, D.S.; Felip, E.; Chow, L.Q.; Camidge, D.R.; Vansteenkiste, J.; Sharma, S.; De Pas, T.; et al. Ceritinib in ALK-rearranged non-small-cell lung cancer. *N Engl J Med* **2014**, *370*, 1189-1197.
9. Jhaveri, K.; Modi, S. Ganetespib: research and clinical development. *Onco Targets Ther* **2015**, *8*, 1849-1858.
10. Fassnacht, M.; Berruti, A.; Baudin, E.; Demeure, M.J.; Gilbert, J.; Haak, H.; Kroiss, M.; Quinn, D.I.; Hesseltine, E.; Ronchi, C.L.; et al. Linsitinib (OSI-906) versus placebo for patients with locally advanced or metastatic adrenocortical carcinoma: A double-blind, randomised, phase 3 study. *Lancet Oncol* **2015**, *16*, 426-435.
11. Kim, D.W.; Mehra, R.; Tan, D.S.W.; Felip, E.; Chow, L.Q.M.; Camidge, D.R.; Vansteenkiste, J.; Sharma, S.; De Pas, T.; Riely, G.J.; et al. Activity and safety of ceritinib in patients with ALK-rearranged non-small-cell lung cancer (ASCEND-1): updated results from the multicentre, open-label, phase 1 trial. *Lancet Oncol* **2016**, *17*, 452-463.
12. Soria, J.C.; Tan, D.S.W.; Chiari, R.; Wu, Y.L.; Paz-Ares, L.; Wolf, J.; Geater, S.L.; Orlov, S.; Cortinovis, D.; Yu, C.J.; et al. First-line ceritinib versus platinum-based chemotherapy in advanced ALK-rearranged non-small-cell lung cancer (ASCEND-4): a randomised, open-label, phase 3 study. *Lancet* **2017**, *389*, 917-929.
13. Nishio, M.; Felip, E.; Orlov, S.; Park, K.; Yu, C.J.; Tsai, C.M.; Cobo, M.; McKeage, M.; Su, W.C.; Mok, T.; et al. Final overall survival and other efficacy and safety results from ASCEND-3: Phase II study of ceritinib in ALKi-naive patients with ALK-rearranged NSCLC. *J Thorac Oncol* **2020**, *15*, 609-617.
14. Chow, L.Q.M.; Barlesi, F.; Bertino, E.M.; van den Bent, M.J.; Wakelee, H.A.; Wen, P.Y.; Chiu, C.H.; Orlov, S.; Chiari, R.; Majem, M.; et al. ASCEND-7: Efficacy and safety of ceritinib treatment in patients with ALK-positive non-small cell lung cancer metastatic to the brain and/or leptomeninges. *Clin Cancer Res* **2022**, *28*, 2506-2516.

15. Fischer, M.; Moreno, L.; Ziegler, D.S.; Marshall, L.V.; Zwaan, C.M.; Irwin, M.S.; Casanova, M.; Sabado, C.; Wulff, B.; Stegert, M.; et al. Ceritinib in paediatric patients with anaplastic lymphoma kinase-positive malignancies: an open-label, multicentre, phase 1, dose-escalation and dose-expansion study. *Lancet Oncol* **2021**, *22*, 1764-1776.
16. Hida, T.; Satouchi, M.; Nakagawa, K.; Seto, T.; Matsumoto, S.; Kiura, K.; Nokihara, H.; Murakami, H.; Tokushige, K.; Hatano, B.; et al. Ceritinib in patients with advanced, crizotinib-treated, anaplastic lymphoma kinase-rearranged NSCLC: Japanese subset. *Jpn J Clin Oncol* **2017**, *47*, 618-624.
17. Kiura, K.; Imamura, F.; Kagamu, H.; Matsumoto, S.; Hida, T.; Nakagawa, K.; Satouchi, M.; Okamoto, I.; Takenoyama, M.; Fujisaka, Y.; et al. Phase 3 study of ceritinib vs chemotherapy in ALK-rearranged NSCLC patients previously treated with chemotherapy and crizotinib (ASCEND-5): Japanese subset. *Jpn J Clin Oncol* **2018**, *48*, 367-375.
18. Ferrarotto, R.; William, W.N.; Tseng, J.E.; Marur, S.; Shin, D.M.; Murphy, B.; Cohen, E.E.W.; Thomas, C.Y.; Willey, R.; Cosaert, J.; et al. Randomized phase II trial of cixutumumab alone or with cetuximab for refractory recurrent/metastatic head and neck squamous cell carcinoma. *Oral Oncol* **2018**, *82*, 83-90.
19. Chugh, R.; Griffith, K.A.; Davis, E.J.; Thomas, D.G.; Zavala, J.D.; Metko, G.; Brockstein, B.; Undevia, S.D.; Stadler, W.M.; Schuetze, S.M. Doxorubicin plus the IGF-1R antibody cixutumumab in soft tissue sarcoma: a phase I study using the TITE-CRM model. *Ann Oncol* **2015**, *26*, 1459-1464.
20. Dasari, A.; Phan, A.; Gupta, S.; Rashid, A.; Yeung, S.C.; Hess, K.; Chen, H.; Tarco, E.; Wei, C.; Anh-Do, K.; et al. Phase I study of the anti-IGF1R antibody cixutumumab with everolimus and octreotide in advanced well-differentiated neuroendocrine tumors. *Endocr Relat Cancer* **2015**, *22*, 431-441.
21. Schwartz, G.K.; Tap, W.D.; Qin, L.X.; Livingston, M.B.; Undevia, S.D.; Chmielowski, B.; Agulnik, M.; Schuetze, S.M.; Reed, D.R.; Okuno, S.H.; et al. Cixutumumab and temsirolimus for patients with bone and soft-tissue sarcoma: a multicentre, open-label, phase 2 trial. *Lancet Oncol* **2013**, *14*, 371-382.
22. Wilky, B.A.; Rudek, M.A.; Ahmed, S.; Laheru, D.A.; Cosgrove, D.; Donehower, R.C.; Nelkin, B.; Ball, D.; Doyle, L.A.; Chen, H.; et al. A phase I trial of vertical inhibition of IGF signalling using cixutumumab, an anti-IGF-1R antibody, and selumetinib, an MEK 1/2 inhibitor, in advanced solid tumours. *Br J Cancer* **2015**, *112*, 24-31.
23. Lerario, A.M.; Worden, F.P.; Ramm, C.A.; Hesseltine, E.A.; Hasseltine, E.A.; Stadler, W.M.; Else, T.; Shah, M.H.; Agamah, E.; Rao, K.; et al. The combination of insulin-like growth factor receptor 1 (IGF1R) antibody cixutumumab and mitotane as a first-line therapy for patients with recurrent/metastatic adrenocortical carcinoma: a multi-institutional NCI-sponsored trial. *Horm Cancer* **2014**, *5*, 232-239.
24. Argiris, A.; Lee, J.W.; Stevenson, J.; Sulecki, M.G.; Hugec, V.; Choong, N.W.; Saltzman, J.N.; Song, W.; Hansen, R.M.; Evans, T.L.; et al. Phase II randomized trial of carboplatin, paclitaxel, bevacizumab with or without cixutumumab (IMC-A12) in patients with advanced non-squamous, non-small-cell lung cancer: a trial of the ECOG-ACRIN Cancer Research Group (E3508). *Ann Oncol* **2017**, *28*, 3037-3043.
25. Yu, E.Y.; Li, H.; Higano, C.S.; Agarwal, N.; Pal, S.K.; Alva, A.; Heath, E.I.; Lam, E.T.; Gupta, S.; Lilly, M.B.; et al. SWOG S0925: A Randomized phase II study of androgen deprivation combined with cixutumumab versus androgen deprivation alone in patients with new metastatic hormone-sensitive prostate cancer. *J Clin Oncol* **2015**, *33*, 1601-1608.
26. Higano, C.S.; Berlin, J.; Gordon, M.; LoRusso, P.; Tang, S.; Dontabhaktuni, A.; Schwartz, J.D.; Cosaert, J.; Mehnert, J.M. Safety, tolerability, and pharmacokinetics of single and multiple doses of intravenous cixutumumab (IMC-A12), an inhibitor of the insulin-like growth factor-I receptor, administered weekly or every 2 weeks in patients with advanced solid tumors. *Invest New Drugs* **2015**, *33*, 450-462.

27. Hussain, M.; Rathkopf, D.; Liu, G.; Armstrong, A.; Kelly, W.K.; Ferrari, A.; Hainsworth, J.; Joshi, A.; Hozak, R.R.; Yang, L.; et al. A randomised non-comparative phase II trial of cixutumumab (IMC-A12) or ramucirumab (IMC-1121B) plus mitoxantrone and prednisone in men with metastatic docetaxel-pretreated castration-resistant prostate cancer. *Eur J Cancer* **2015**, *51*, 1714-1724.
28. Rugo, H.S.; Trédan, O.; Ro, J.; Morales, S.M.; Campone, M.; Musolino, A.; Afonso, N.; Ferreira, M.; Park, K.H.; Cortes, J.; et al. A randomized phase II trial of ridaforolimus, dalotuzumab, and exemestane compared with ridaforolimus and exemestane in patients with advanced breast cancer. *Breast Cancer Res Treat* **2017**, *165*, 601-609.
29. Ellis, P.M.; Shepherd, F.A.; Laurie, S.A.; Goss, G.D.; Olivo, M.; Powers, J.; Seymour, L.; Bradbury, P.A. NCIC CTG IND.190 phase I trial of dalotuzumab (MK-0646) in combination with cisplatin and etoposide in extensive-stage small-cell lung cancer. *J Thorac Oncol* **2014**, *9*, 410-413.
30. Huang, C.H.; Williamson, S.K.; Neupane, P.; Taylor, S.A.; Allen, A.; Smart, N.J.; Uypeckcuat, A.M.; Spencer, S.; Wick, J.; Smith, H.; et al. Impact study: MK-0646 (dalotuzumab), insulin growth factor 1 receptor antibody combined with pemetrexed and cisplatin in stage IV metastatic non-squamous lung cancer. *Front Oncol* **2015**, *5*, 301.
31. Brana, I.; Berger, R.; Golan, T.; Haluska, P.; Edenfield, J.; Fiorica, J.; Stephenson, J.; Martin, L.P.; Westin, S.; Hanjani, P.; et al. A parallel-arm phase I trial of the humanised anti-IGF-1R antibody dalotuzumab in combination with the AKT inhibitor MK-2206, the mTOR inhibitor ridaforolimus, or the NOTCH inhibitor MK-0752, in patients with advanced solid tumours. *Br J Cancer* **2014**, *111*, 1932-1944.
32. Sclafani, F.; Kim, T.Y.; Cunningham, D.; Kim, T.W.; Tabernero, J.; Schmoll, H.J.; Roh, J.K.; Kim, S.Y.; Park, Y.S.; Guren, T.K.; et al. A randomized phase 2/3 study of dalotuzumab in combination with cetuximab and irinotecan in chemorefractory, KRAS wild-type, metastatic colorectal cancer. *J Natl Cancer Inst* **2015**, *107*, djv258.
33. Moran, T.; Felip, E.; Keedy, V.; Borghaei, H.; Shepherd, F.A.; Insa, A.; Brown, H.; Fitzgerald, T.; Sathyanarayanan, S.; Reilly, J.F.; et al. Activity of dalotuzumab, a selective anti-IGF1R antibody, in combination with erlotinib in unselected patients with Non-small-cell lung cancer: a phase I/II randomized trial. *Exp Hematol Oncol* **2014**, *3*, 26.
34. Becerra, C.R.; Salazar, R.; Garcia-Carbonero, R.; Thomas, A.L.; Vázquez-Mazón, F.J.; Cassidy, J.; Maughan, T.; Castillo, M.G.; Iveson, T.; Yin, D.; et al. Figitumumab in patients with refractory metastatic colorectal cancer previously treated with standard therapies: a nonrandomized, open-label, phase II trial. *Cancer Chemother Pharmacol* **2014**, *73*, 695-702.
35. Langer, C.J.; Novello, S.; Park, K.; Krzakowski, M.; Karp, D.D.; Mok, T.; Benner, R.J.; Scranton, J.R.; Olszanski, A.J.; Jassem, J. Randomized, phase III trial of first-line figitumumab in combination with paclitaxel and carboplatin versus paclitaxel and carboplatin alone in patients with advanced non-small-cell lung cancer. *J Clin Oncol* **2014**, *32*, 2059-2066.
36. Yin, D.; Sleight, B.; Alvey, C.; Hansson, A.G.; Bello, A. Pharmacokinetics and pharmacodynamics of figitumumab, a monoclonal antibody targeting the insulin-like growth factor 1 receptor, in healthy participants. *J Clin Pharmacol* **2013**, *53*, 21-28.
37. Scagliotti, G.V.; Bondarenko, I.; Blackhall, F.; Barlesi, F.; Hsia, T.C.; Jassem, J.; Milanowski, J.; Popat, S.; Sanchez-Torres, J.M.; Novello, S.; et al. Randomized, phase III trial of figitumumab in combination with erlotinib versus erlotinib alone in patients with nonadenocarcinoma nonsmall-cell lung cancer. *Ann Oncol* **2015**, *26*, 497-504.
38. Olmos, D.; Postel-Vinay, S.; Molife, L.R.; Okuno, S.H.; Schuetze, S.M.; Paccagnella, M.L.; Batzel, G.N.; Yin, D.; Pritchard-Jones, K.; Judson, I.; et al. Safety, pharmacokinetics, and preliminary activity of the anti-IGF-1R antibody figitumumab (CP-751,871) in patients with sarcoma and Ewing's sarcoma: a phase 1 expansion cohort study. *Lancet Oncol* **2010**, *11*, 129-135.

39. Meehan, R.; Kummar, S.; Do, K.; O'Sullivan Coyne, G.; Juwara, L.; Zlott, J.; Rubinstein, L.; Doroshow, J.H.; Chen, A.P. A phase I study of ganetespib and ziv-aflibercept in patients with advanced carcinomas and sarcomas. *Oncologist* **2018**, *23*, 1269-e1125.
40. Strosberg, J.R.; Chan, J.A.; Ryan, D.P.; Meyerhardt, J.A.; Fuchs, C.S.; Abrams, T.; Regan, E.; Brady, R.; Weber, J.; Campos, T.; et al. A multi-institutional, phase II open-label study of ganitumab (AMG 479) in advanced carcinoid and pancreatic neuroendocrine tumors. *Endocr Relat Cancer* **2013**, *20*, 383-390.
41. Smith, T.J.; Kahaly, G.J.; Ezra, D.G.; Fleming, J.C.; Dailey, R.A.; Tang, R.A.; Harris, G.J.; Antonelli, A.; Salvi, M.; Goldberg, R.A.; et al. Teprotumumab for thyroid-associated ophthalmopathy. *N Engl J Med* **2017**, *376*, 1748-1761.
42. Xin, Y.; Xu, F.; Gao, Y.; Bhatt, N.; Chamberlain, J.; Sile, S.; Hammel, S.; Holt, R.J.; Ramanathan, S. Pharmacokinetics and exposure-response relationship of teprotumumab, an insulin-like growth factor-1 receptor-blocking antibody, in thyroid eye disease. *Clin Pharmacokinet* **2021**, *60*, 1029-1040.
43. Sears, C.M.; Azad, A.D.; Amarikwa, L.; Pham, B.H.; Men, C.J.; Kaplan, D.N.; Liu, J.; Hoffman, A.R.; Swanson, A.; Alyono, J.; et al. Hearing dysfunction after treatment with teprotumumab for thyroid eye disease. *Am J Ophthalmol* **2022**, *240*, 1-13.
44. Ramalingam, S.S.; Spigel, D.R.; Chen, D.; Steins, M.B.; Engelman, J.A.; Schneider, C.P.; Novello, S.; Eberhardt, W.E.; Crino, L.; Habben, K.; et al. Randomized phase II study of erlotinib in combination with placebo or R1507, a monoclonal antibody to insulin-like growth factor-1 receptor, for advanced-stage non-small-cell lung cancer. *J Clin Oncol* **2011**, *29*, 4574-4580.
45. United States Food and Drug Administration. Center for Drug Evaluation and Research. Application number: 761143Orig1s000 Summary Review [Teprezza]. Available online: https://www.accessdata.fda.gov/drugsatfda_docs/nda/2021/761143Orig1s000SumR.pdf (accessed on February 22, 2023).
